# Multi-target combination treatment with rTMS and tDCS for Tourette syndrome: a case report

**DOI:** 10.3389/fnhum.2024.1441019

**Published:** 2025-01-07

**Authors:** Shuang Zhao, Qizu Jin, Qi Yang, Jing Liu, Yun Lu, Haibo Ai

**Affiliations:** ^1^Hospital of Chengdu University of Traditional Chinese Medicine, Chengdu, China; ^2^Clinical Medical School, Chengdu University of Traditional Chinese Medicine, Chengdu, China; ^3^The Third Hospital of Mianyang, Sichuan Mental Health Center, Mianyang, China

**Keywords:** Tourette syndrome, repetitive transcranial magnetic stimulation, rTMS, transcranial direct current stimulation, tDCS

## Abstract

Tourette syndrome (TS) is a neuropsychiatric disorder characterized by chronic motor and phonic tics, with a higher prevalence among boys. This condition can significantly impact patients’ learning and daily life. Due to the limited efficacy and potential side effects of pharmacological treatments for TS, there is a critical need to develop novel, tailored therapeutic strategies. Repetitive transcranial magnetic stimulation (rTMS) and transcranial direct current stimulation (tDCS) have been proposed as potential treatments for TS, and have shown promising results. Here, we report a case of refractory TS, in which low-frequency rTMS was delivered to the left supplementary motor area (SMA), combined with tDCS targeting the primary motor cortex (M1) and the cerebellum, with the cathode positioned over the right M1 and the anode over the left cerebellum. This is the first reported case using a multi-target combination therapy for TS. This treatment yielded favorable outcomes and maintained good efficacy during a three-month follow-up period. Although larger-scale trials are needed, our findings pave the way for the application of non-invasive brain stimulation techniques in TS, offering a transformative path to improve treatment outcomes and quality of life for those with TS.

## Introduction

Tourette syndrome (TS) manifests as various motor tics and at least one phonic tic lasting more than 1 year (Pringsheim et al., 2019), and is more common in boys (American Psychiatric Association, 2013). This condition can significantly affect both the physiological and psychological development of the patient. Although TS is currently managed through medication and behavioral therapy, side effects and limited access to specialized resources may pose challenges to effective treatment. Therefore, it is essential to explore new treatment methods that are less prone to side effects and more easily accessible.

Recently, non-invasive brain stimulation techniques such as repetitive transcranial magnetic stimulation (rTMS) and transcranial direct current stimulation (tDCS) have emerged as alternative therapies for TS (Kahl et al., 2021; Dyke et al., 2022). In addition, the safety of these two methods in children and adolescents has been demonstrated (Krishnan et al., 2015).

The supplementary motor area (SMA) is considered a preferred target for rTMS or tDCS for the treatment of TS due to its involvement in the pathomechanisms of TS (Kleimaker et al., 2020; Yu et al., 2022). Functional neuroimaging studies in humans and experimental investigations in animals have demonstrated that the onset of tics in TS is characterized by complex interactions among cortical-striatal-thalamic-cortical (CSTC) brain circuits (Sigurdsson et al., 2020). SMA plays an important role in TS through CSTC brain circuits (Yu et al., 2022). In TS, the excitability of SMA is increased due to the abnormal activity of specific striatal neuronal subpopulations, leading to disinhibition of thalamocortical projections (Albin and Mink, 2006; Hashemiyoon et al., 2017; Yael et al., 2015). Meanwhile, SMA serves as the primary target for basal ganglia projections (Akkal et al., 2007) and is likely to drive M1 and enhance its activation (Franzkowiak et al., 2012). Therefore, inhibiting SMA excitability is key to treating TS (Hsu et al., 2018). Studies indicated that both rTMS and tDCS can modulate SMA excitability via conditional stimulation. For instance, 1 Hz low-frequency rTMS over SMA can reduce the excitability of this region, significantly decrease tic severity in TS, and exhibit a cumulative effect (Hsu et al., 2018). Cathodal tDCS over SMA can reduce the frequency and intensity of tics in patients with TS (Eapen et al., 2017).

In addition to CSTC brain circuits, the dentato-thalamo-cortical (DTC) pathway appears to be involved in the development of tics in TS (Tremblay et al., 2016). The cerebellum plays a crucial role in the regulation of motor control and is connected to M1 through the DTC pathway (Ates et al., 2018). During the tic process in TS, the latencies of pathological activity in the cerebellum and M1 overlapped significantly, suggesting that aberrant signals may travel along divergent pathways to these structures from the basal ganglia (McCairn et al., 2013). In TS, M1 excitability is increased, and reduction of this excitability is often linked with better control of the tics (Franzkowiak et al., 2012). Meanwhile, research on other neuropsychiatric disorders, such as Obsessive-Compulsive Disorder (OCD) has indicated that applying cathodal tDCS or low-frequency rTMS over M1 can decrease the excitability of M1 (Lefaucheur et al., 2014; Stagg et al., 2018) and the application of anodal tDCS over cerebellum can reduce the excitability of contralateral M1 through cerebello-brain inhibition (CBI) (Schlerf et al., 2015; Tremblay et al., 2016; Ates et al., 2018; Ugawa and Manto, 2023). However, TMS or tDCS over cerebellum or M1 region has not been conducted in TS patients. Therefore, according to the pathophysiologic mechanisms of TS, we tried to apply rTMS or tDCS over M1 or cerebellum in TS patients, hoping the multi-targets combined treatment is effective for TS patients.

**Figure 1:**
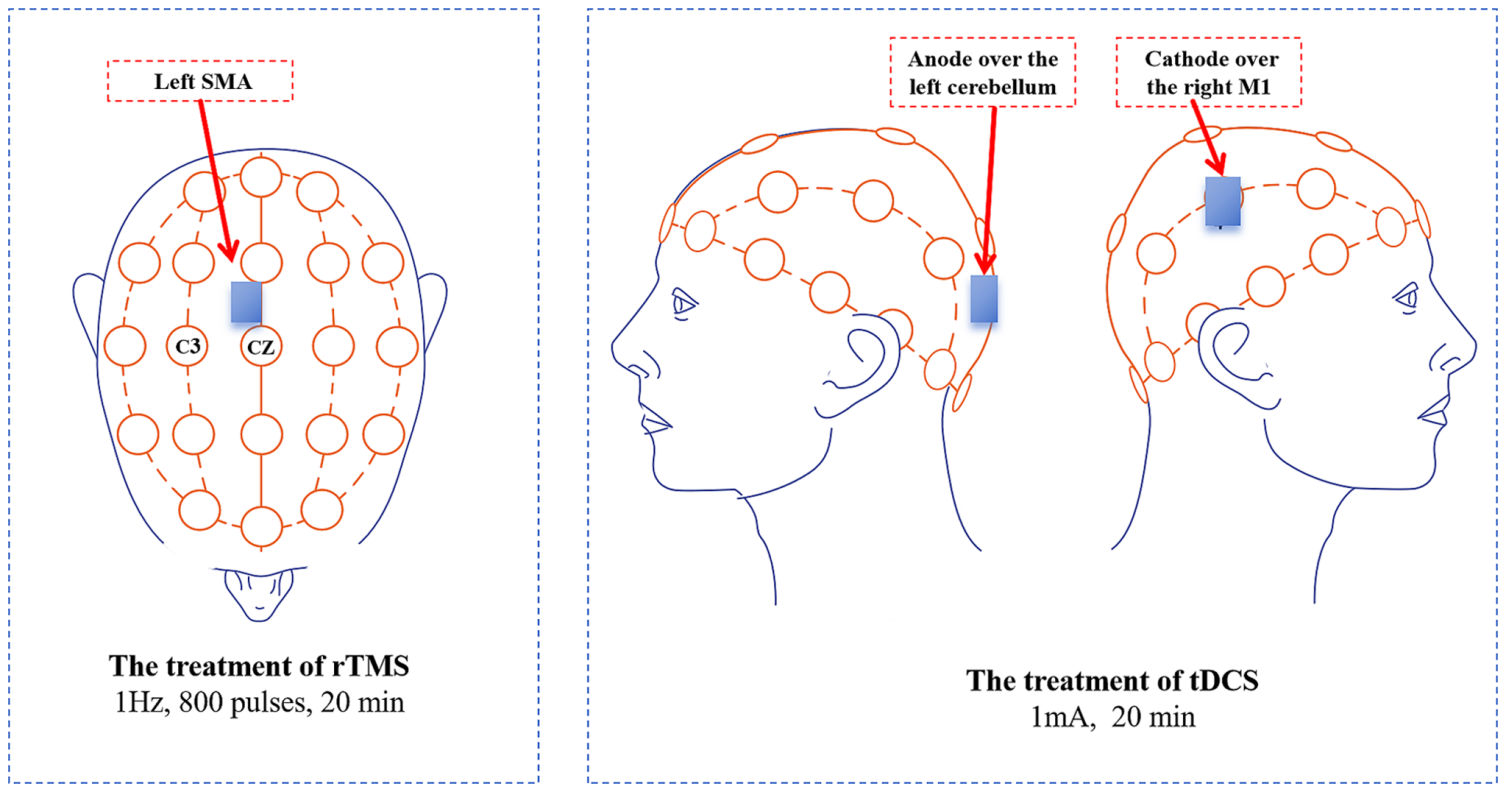
Treatment targets and methods in this case. tDCS, Transcranial direct current stimulation; rTMS, Repetitive transcranial magnetic stimulation; SMA, Supplementary motor area.

In this study, we selected three stimulation targets: SMA, M1, and the cerebellum, based on the pathogenesis of TS. We applied low-frequency rTMS over SMA. Given the overlapping latency periods of pathological activity between the cerebellum and M1 (McCairn et al., 2013), we chose to stimulate these two targets simultaneously with tDCS (Figure 1). A single session of rTMS or tDCS produces a rapid effect, but it is short-lived. Cumulative effects can occur with multiple treatments (Lefaucheur, 2009; Stagg et al., 2018). Therefore, we set a 10-session treatment cycle to achieve a cumulative effect. We hypothesized that this multi-target combination treatment would be effective in TS and capable of sustaining therapeutic effects. This is the first report of multi-target combination treatment with rTMS and tDCS for TS, aiming to explore a therapeutic regimen with long-lasting efficacy, easy accessibility, minimal side effects, and a high degree of safety, thereby offering valuable insights into TS treatment.

## Case presentation

In July 2021, an 11-year-old boy with refractory TS presented to our hospital. He had been experiencing involuntary head and neck tics, as well as abnormal throat sounds, for 4 years prior to his visit, with each episode lasting from 10 to 30 s. These symptoms intensified during periods of emotional excitement and significantly impacted his learning and daily life. He had no comorbidities, such as obsessive-compulsive disorder or attention deficit hyperactivity disorder (ADHD). His brain MRI and electroencephalogram (EEG) results were normal. There was no family history of TS or tics. Over the preceding years, he had undergone long-term treatment with various medications, including antipsychotic drugs (Tiapride 0.05 g three times daily), antiepileptic drugs (Gabapentin 0.1 g three times daily), and centrally acting skeletal muscle relaxants (Tizanidine 1 mg three times daily). However, his clinical response to these treatments was poor. Given the chronic nature of the disease and the significant social consequences associated with the disorder, we decided to propose a combination treatment of rTMS and tDCS. Before the treatment, the patient and his family were informed about the purpose and procedure of the treatment, and written informed consent was obtained.

**Figure 2:**
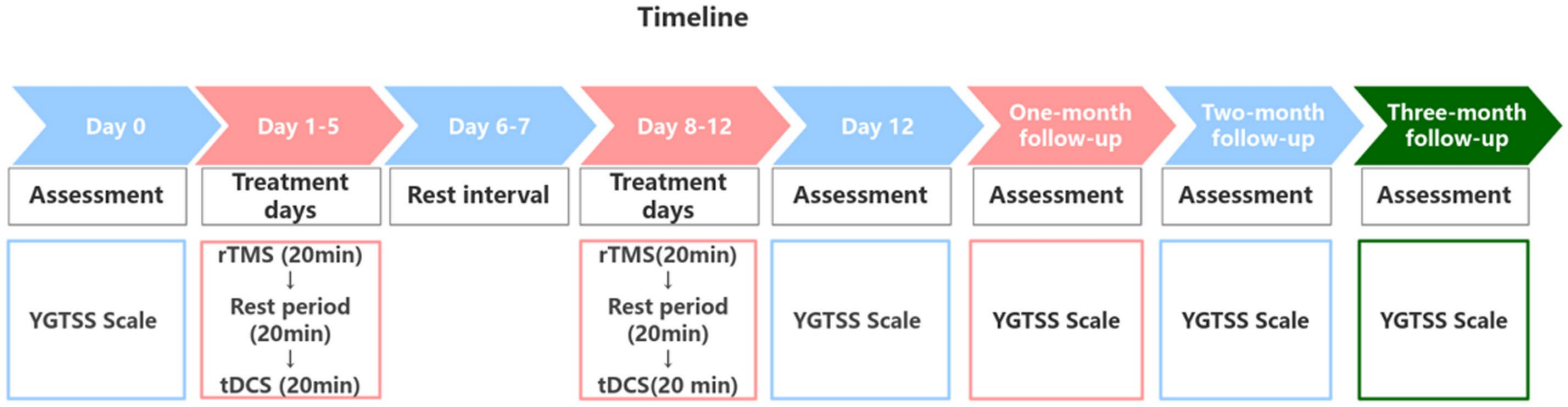
Treatment and the follow-up timeline.

The entire protocol lasted 12 days and included 10 treatment sessions. It began with 5 consecutive days of treatment, followed by a 2-day rest interval, and concluded with another 5 days of treatment. The patient’s 10 treatment sessions were conducted in the hospital by the specialized rehabilitation therapists. Each treatment day commenced with low-frequency rTMS administered over the left SMA, followed by a 20-min rest period, after which tDCS was applied over the right M1 and the left cerebellum (Figure 2). Prior to treatment, the contraindications for rTMS and tDCS were screened in the patient, including the presence of implanted metal devices, intracranial infections, epilepsy, severe heart disease, and skin damage at the stimulation site and so on. The detailed treatment protocols were as follows:

### The treatment of rTMS

This treatment was conducted using AIM-III magnetic robot (Wuhan Zilian Hongkang Technology Co., Ltd., China). First, we performed a resting motor threshold (RMT) measurement. The patient sat quietly in a chair while the therapist assessed the RMT for both the left and right cerebral hemispheres. In a relaxed state, the motor cortex (MC) area corresponding to the abductor pollicis brevis muscle was stimulated to identify the optimal stimulation point that elicited maximum amplitude and repeatable motor evoked potentials (MEP), resulting in contraction of the contralateral abductor pollicis brevis muscle. The output intensity was finely adjusted until the minimum stimulation intensity that produced MEPs greater than 50 μV in over 5 out of 10 consecutive TMS pulses was established (Huang et al., 2018). After completing the RMT measurement, we identified the left SMA according to the international 10–20 system electrode placement method. This target is positioned at 15% of the distance from the nasion (the point between the eyebrows) to the external occipital protuberance (the most prominent point on the back of the head) and is located anterior to Cz (the midpoint of the line connecting the nasion and the external occipital protuberance). The AIM-III employed a spatial localization algorithm to map SMA to the corresponding position on the head model, inputting the spatial location into the AIM magnetic stimulation robot system in real-time. This ensured precise targeting as the system controlled the coil to reach the treatment target. Once accurate localization was achieved, treatment commenced with a stimulation frequency of 1 Hz, a stimulation intensity of 80% RMT, and consisted of 10 s of stimulation followed by a 5-s interval (De Vito et al., 2009), totaling 800 pulses over a 20-min session. After the first treatment, the patient’s records were saved, enabling retrieval of preset treatment plans for subsequent sessions, eliminating the need for reconfiguration.

### The treatment of tDCS

We selected the right M1 and the left cerebellum for tDCS treatment. A portable tDCS device (EM600, Wuhan Yimai Medical Technology Co., Ltd., China) was used for this treatment. The M1 location was identified according to the international 10–20 system electrode placement method, specifically at the C3/C4 regions. We used saline-soaked sponges to improve the quality of contact during the tDCS treatment. Each sponge measured 5 cm × 7 cm. The cathode was placed over the right M1, while the anode was placed over the left cerebellum, then delivering a treatment current of 1 mA based on previous study (Ferrucci et al., 2015). The duration of the treatment was 20 min.

### Behavioral evaluation with YGTSS score

The Yale Global Tic Severity Scale (YGTSS) score (Leckman et al., 1989) was employed to assess the patient’s condition at five key time points: before treatment (Day 0), the day after treatment concluded (Day 12) and during the one-month, two-month, and three-month follow-ups. This scale assesses both motor and phonic tics (including number, frequency, intensity, complexity, and interference), with each category rated on a scale from 0 to 25. The total YGTSS score is derived from the sum of the motor tic score and the phonic tic score. In this study, we recorded the motor, phonic and the total tic scores.

## Results

**Figure 3:**
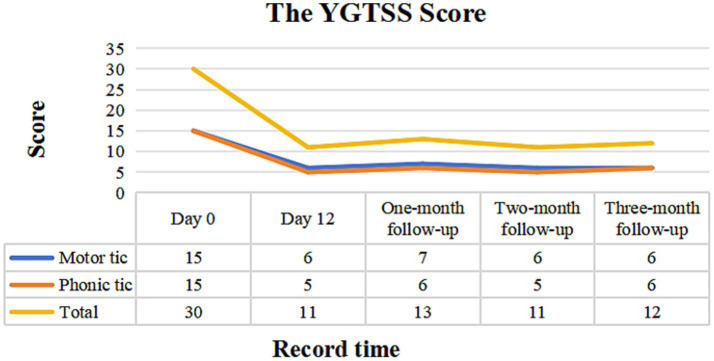
YGTSS scale assessment before treatment, at the end of treatment and at the subsequent three-month follow-ups visit. YGTSS, Yale Global Tic Severity Scale.

After four treatment sessions, both the frequency and intensity of motor and phonic tics were reduced in the patient. Also, our patient exhibited a good tolerance to the treatment, without obvious adverse events. During the follow-up period, the child’s learning and daily life were minimally affected by TS, with no significant deterioration. The total YGTSS score decreased from 30 at baseline to 11 on the day of treatment completion (Day 12), a reduction of 63.3%. Specifically, the motor tics score decreased from 15 to 6, a 60.0% reduction, and the phonic tics score decreased from 15 to 5, a 66.7% reduction. During the first month of follow-up, the total YGTSS score was 13, with motor tics scored at 7 and phonic tics at 6. In the second month of follow-up, the total YGTSS score remained at 11, with motor tics at 6 and phonic tics at 5. By the third month of follow-up, the total YGTSS score was 12, with both motor and vocal tics recorded at 6 (Figure 3).

## Discussion

This study suggests that 10 sessions of multi-targeted combination therapy with rTMS and tDCS may be effective for children with TS, and significant effects on motor and phonic tics were observed. The effects of this treatment were sustained for up to 3 months and were comparable to those observed in previous studies using low-frequency rTMS superior to SMA. However, the duration of treatment was shorter than conventional rTMS treatment (Kwon et al., 2011). Therefore, our findings supported that the combination therapy of rTMS and tDCS in TS patients may be a safe and cost-effective treatment regimen.

Although rTMS can facilitate multi-target stimulation (Zheng and Xu, 2020), there is a lack of evidence-based medical support for the effectiveness and safety of multi-target rTMS in treating TS, particularly in children and adolescents. In contrast, studies have shown the safety of using tDCS to stimulate multiple targets simultaneously in pediatric patients with other neurodevelopmental disorders, such as ADHD and autism (Salehinejad et al., 2022). Therefore, we chose to use tDCS for simultaneous stimulation of M1 and the cerebellum. Previous studies have also paved the way for tDCS in treating TS, and mainly applied cathodal tDCS over SMA or pre-SMA, but the findings were mixed (Behler et al., 2018; Carvalho et al., 2015). Hence, we changed the stimulation targets in this study. Consequently, the efficacy was better than that observed in previous study (Carvalho et al., 2015). Thus, we hold that the M1 and the cerebellum might be suitable targets of tDCS for TS and tDCS is a good option for multi-target treatment.

Our study was the first case of a combination treatment with rTMS and tDCS over multiple targets for TS patients. The mechanism of therapeutic action may involve modulation of SMA and M1 activity through various pathways, and the cerebellum may play an crucial role in enhancing M1 inhibition. No significant adverse events or side effects were reported during the entire treatment course and the subsequent 3-month follow-ups. Although our study was a single case report, our successful practice of such therapy protocols may provide strong evidence for the safety and effectiveness of multi-targets non-invasive neurostimulation techniques for TS in the future. Meanwhile, our findings confirmed the potential of an alternative therapy for TS children with symptoms poorly controlled or intolerable of drug therapy, or concerned about the adverse effects of medication. Furthermore, in terms of treatment costs, the tDCS is less expensive than rTMS. So, our treatment protocol could help TS patients with poor economic conditions reduce their medical expenses without diminishing the therapeutic effect. Considering the efficacy, safety, and social benefits of therapeutic regimen, we hoped that our protocol would offer valuable insights for future basic research, clinical practice and application.

Although the results of this study are promising, several limitations must be acknowledged: First, this case report represents only a single case study from China, which requires confirmation with a larger sample across multi-ethnic populations to support our positive findings. Second, randomized, double-blind, sham-controlled studies are needed to verify the effectiveness of the results and improve the level of evidence. Third, the combination of rTMS and tDCS in this study achieved comparable efficacy while requiring shorter treatment durations compared to conventional rTMS treatment. Future studies could further optimize the treatment regimen and directly compare the clinical efficacy of tDCS alone with that of rTMS alone. Last, objective evaluation methods of functional neuroimaging: such as functional near-infrared spectroscopy (fNIRS), functional magnetic resonance imaging (fMRI), and diffusion weighted imaging (DWI) and so on would be considered in our future studies.

## Conclusion

Our study suggests that multi-target combination treatment with rTMS and tDCS is effective and safe for children with TS and can significantly improve motor and phonic tics. The results of our study supported that M1 and the cerebellum may serve as new and effective targets for tDCS in the treatment of TS, further proving that M1 and the cerebellum played an important role in the pathogenesis of TS.

## Data Availability

The raw data supporting the conclusions of this article will be made available by the authors, without undue reservation.
